# Supplementary material for: CSS: cluster similarity spectrum integration of single-cell genomics data
Source: Genome Biol. 2020 Sep 1;21:224. doi: 10.1186/s13059-020-02147-4 (PMC7460789; doi:10.1186/s13059-020-02147-4)
Supplement: Supplementary file 1 — Additional file 1. Supplementary figures. [file 13059_2020_2147_MOESM1_ESM.docx]

**Supplementary figures**


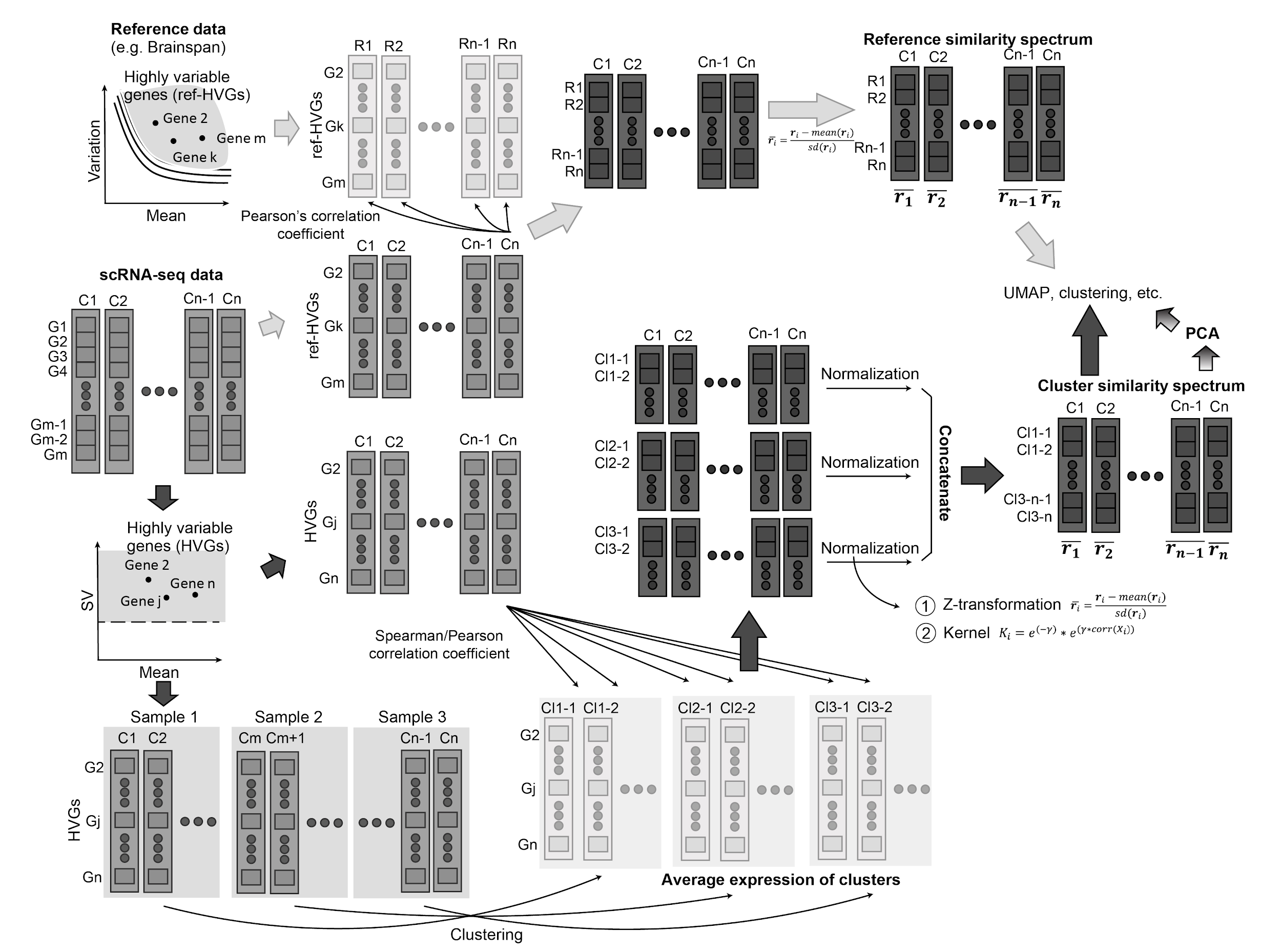


**Fig. S1. Detailed schematic of RSS and CSS representation calculation.** To calculate RSS, highly variable genes in the reference data are first identified. Transcriptome similarity between each cell in the scRNA-seq data is calculated to each of reference samples across the identified genes. The resulting correlations of one cell are normalized using z-transform to obtain its RSS representation. To calculate CSS, clustering is applied to cells in each sample separately. Average transcriptome profiles of clusters in samples are calculated. Similarities between transcriptome of each cell and the cluster average transcriptome profiles are calculated. For each cell, similarities to clusters of one sample are normalized by z-transform or kernel probability transformation, with the normalized similarities to different samples concatenated to obtain its CSS representation. The CSS representation can be directly used as the input of the following analysis, or alternatively, an extra PCA is applied to the CSS representation to further condense the information and reduce the dimensionality.

**
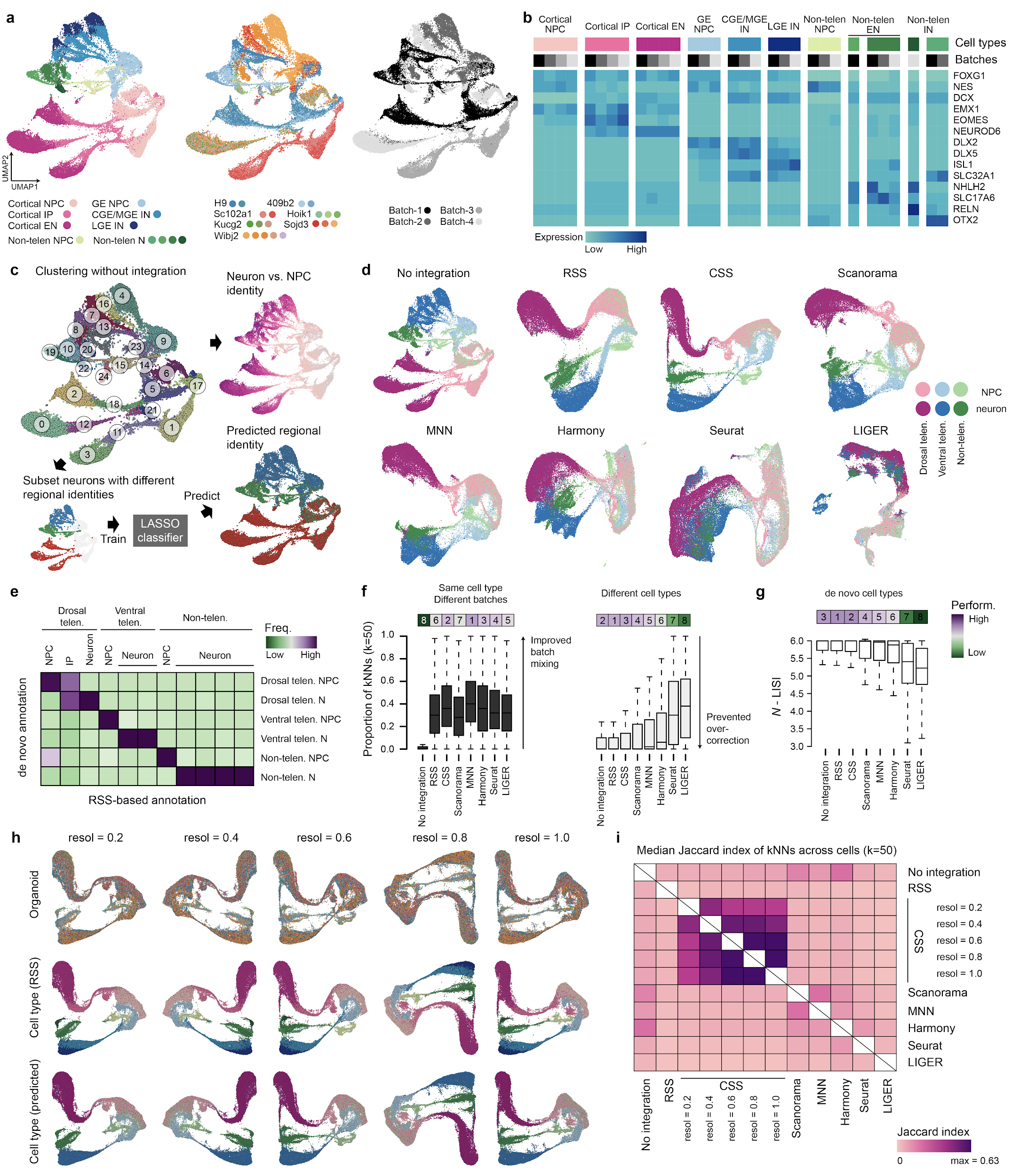
**

**Fig. S2. Cell type annotation of the cerebral organoid scRNA-seq data and CSS robustness to clustering of samples.** (a) Joint analyzing scRNA-seq data of all the twenty cerebral organoids with no integration. The UMAP embedding is colored by RSS-based cell type annotation (left), organoids (middle), and experimental batches (right), respectively. (b) Average expression patterns of selected cell type markers in different cell types, with cells from organoids of different experimental batches separated. (c) Schematic of the model-based prediction of cell type. A random subset of cells in the dorsal/ventral/non- telencephalic neuron clusters were selected to train a prediction model of regional identities. The model was applied to all cells for their predicted regional identities. This information was combined with the NPC/neuron identity estimated by comparing expressions of NPC/neuron markers to get the predicted cell type annotation. (d) UMAP embeddings based on no integration or one of the seven integration methods, colored by the predicted cell type annotation. (e) Heatmap showing consistency between the RSS-derived annotation and the predicted cell type annotation. (f) Proportion of neighbors of each cell which (left) share the same predicted cell type but from a different batch, or (right) are of different predicted cell types. Neighbors are defined as 50 cells with the shortest Euclidean distances with the cell in PCA (no integration) or different integration spaces. Bars on top are colored by the median proportions, with the numbers showing ranks of different methods. (g) LISI-based scores of the model-based cell type annotation with no or different integration methods. Bars on top are colored by the median proportions, with the numbers showing ranks of different methods. (h) UMAP embeddings based on CSS with different clustering resolution used at the step of cell clustering for each sample separately. The embeddings are colored by organoids (upper row), RSS-derived annotation (middle row) and the predicted cell type annotation (bottom row). (i) Pairwise similarity between kNN graphs (k=50) generated using different integration methods with different parameters. The similarity between two kNN graphs is represented as the median Jaccard index of cell neighborhood in the two graphs across all cells.


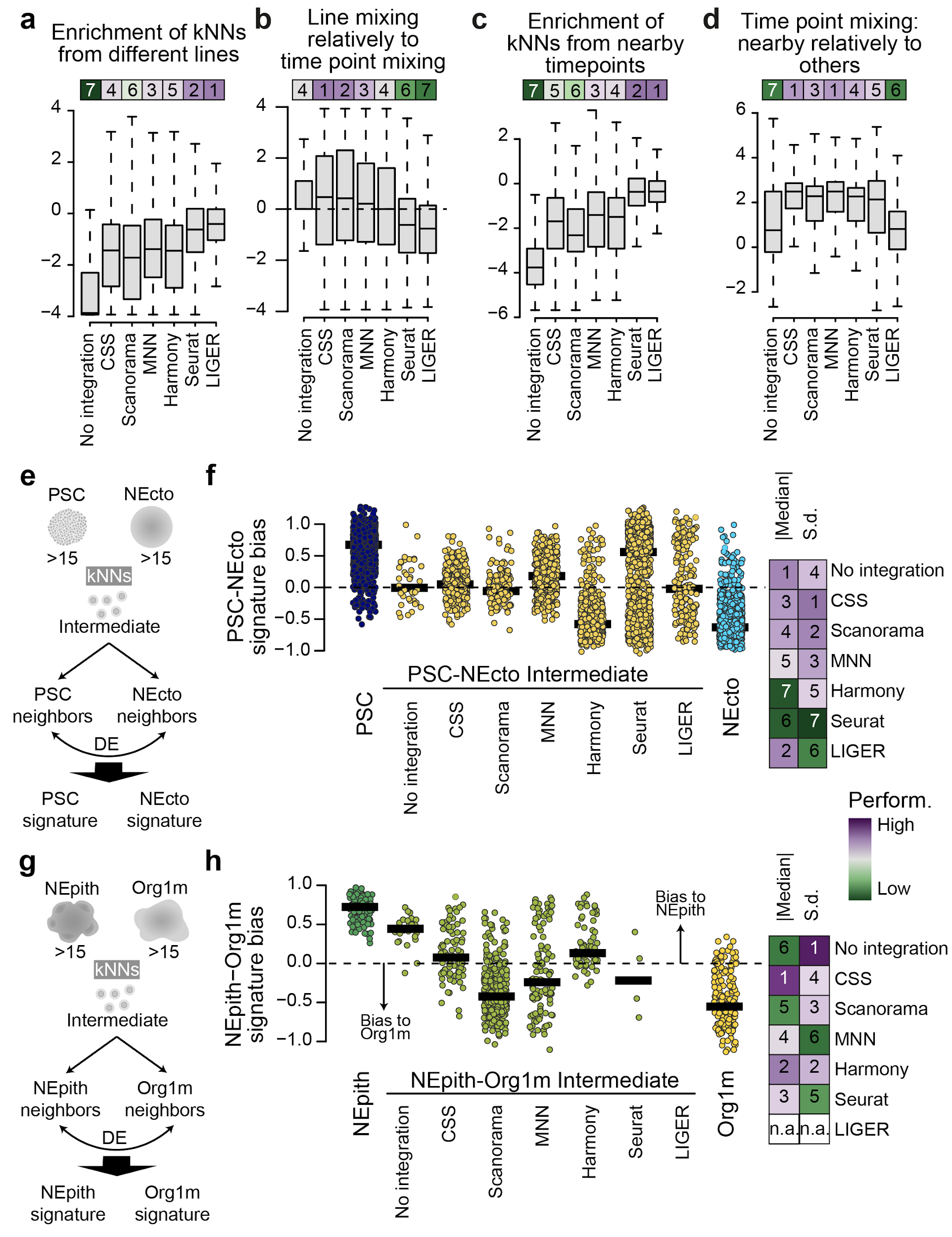


**Fig. S3. Benchmark of different integration methods on the time course cerebral organoid scRNA-seq data set.** (a) For each integration method,boxplots show enrichment of neighboring cells from a different line in comparison to the global proportion of cells from different lines in the whole data set. An increased enrichment indicates improved mixing of cells from different lines. (b) Enrichment of neighboring cells from a different line relative to that of neighboring cells from a different time point. (c) For each integration method, boxplots show enrichment of neighboring cells from nearby time points, in comparison to the global proportion of cells from different time points in the whole data set. An increased enrichment suggests improved connectivity between cells from nearby time points. (d) Enrichment of neighboring cells from nearby time points relative to other time points. A higher relative enrichment suggests better resolving the temporal orders of the biological processes. Bars on top of (a-d) are colored by the median proportions, with the numbers showing ranks of different methods. (e) Schematic of identifying and benchmarking intermediate cells between PSC and neuroectoderm (NEcto) time points. (f) Benchmark of signature unbiasedness of identified PSC-NEcto intermediate cells. The scatter plot shows distributions of PSC-NEcto signature biases of PSC-NEcto intermediate cells identified based on different integration methods. Every dot represents one cell and the lines show the medians. The heatmap shows the summarized scores to rank different integration methods. A good set of intermediate cells is expected to concentrate across zero bias. (g-h) Similar to (e-f) but to identify and benchmark intermediate cells between neuroepithelium (NEpith) and one-month-old organoids (Org1m) time points.


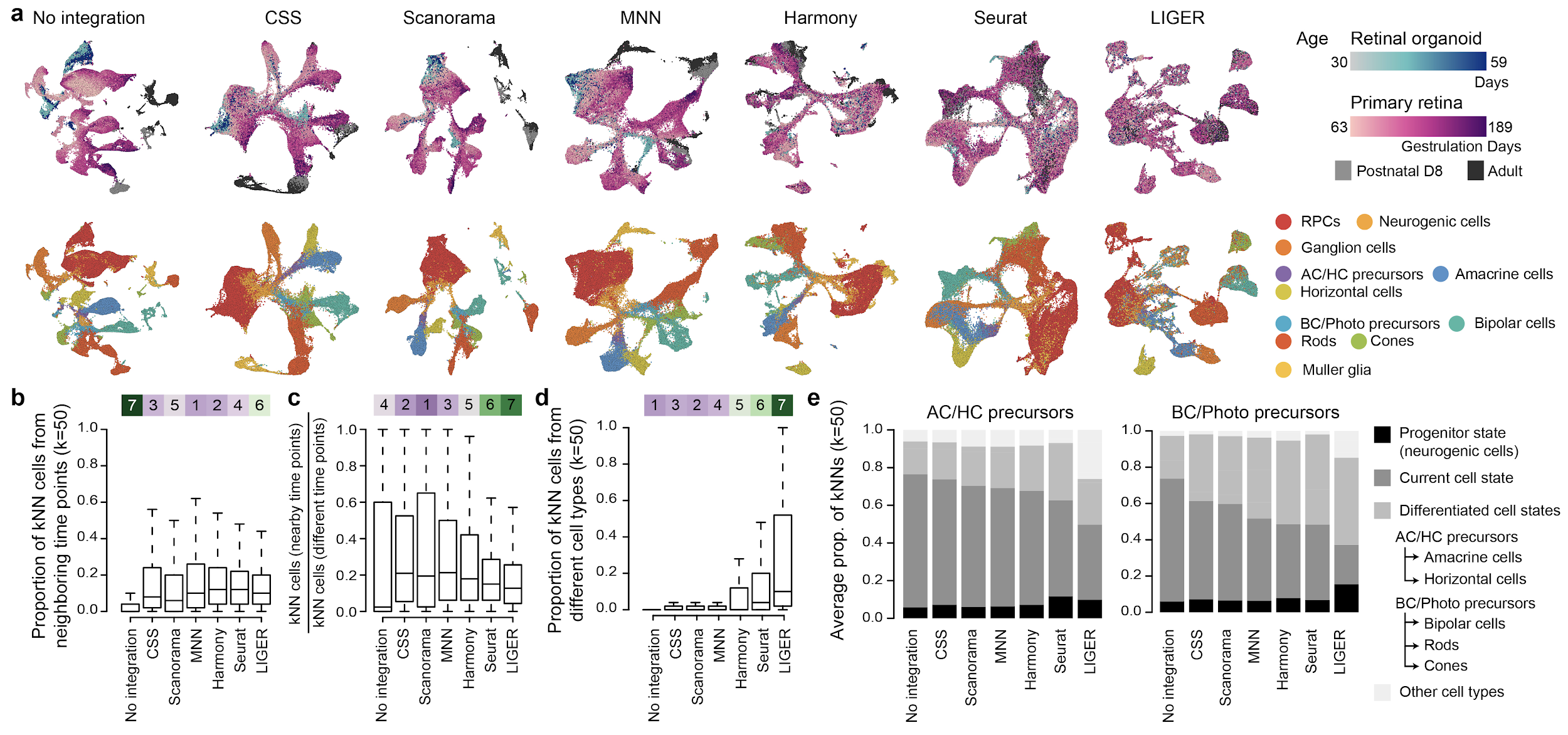


**Fig. S4. Benchmark of different integration methods on the time course human retinal organoid and primary retina scRNA-seq data set.** (a) UMAP embeddings based on no integration or one of the six integration methods, colored by the sample ages (upper) and annotated cell types (lower). (b) Proportion of neighbors of each cell which were from samples at neighboring time points. (c) Proportion of neighbors which were from the neighboring time points among all neighbors which were from any different time point. (d) Proportion of neighbors annotated as different cell types. Neighbors are defined as 50 cells with the shortest Euclidean distances with the cell in PCA (no integration) or different integration spaces. Bars on top of (b-d) are colored by the median proportions, with the numbers showing ranks of different methods. (e) Stacked bar plots showing average proportions of cells annotated as the progenitor cell type (dark), same cell type (grey), differentiated cell type (lighgrey), and others (lightest grey), in the neighborhood of cells annotated as two groups of intermediate precursors: AC/HC precursors (left) and BC/Photo precursors (right), when no integration or different integration methods are used.


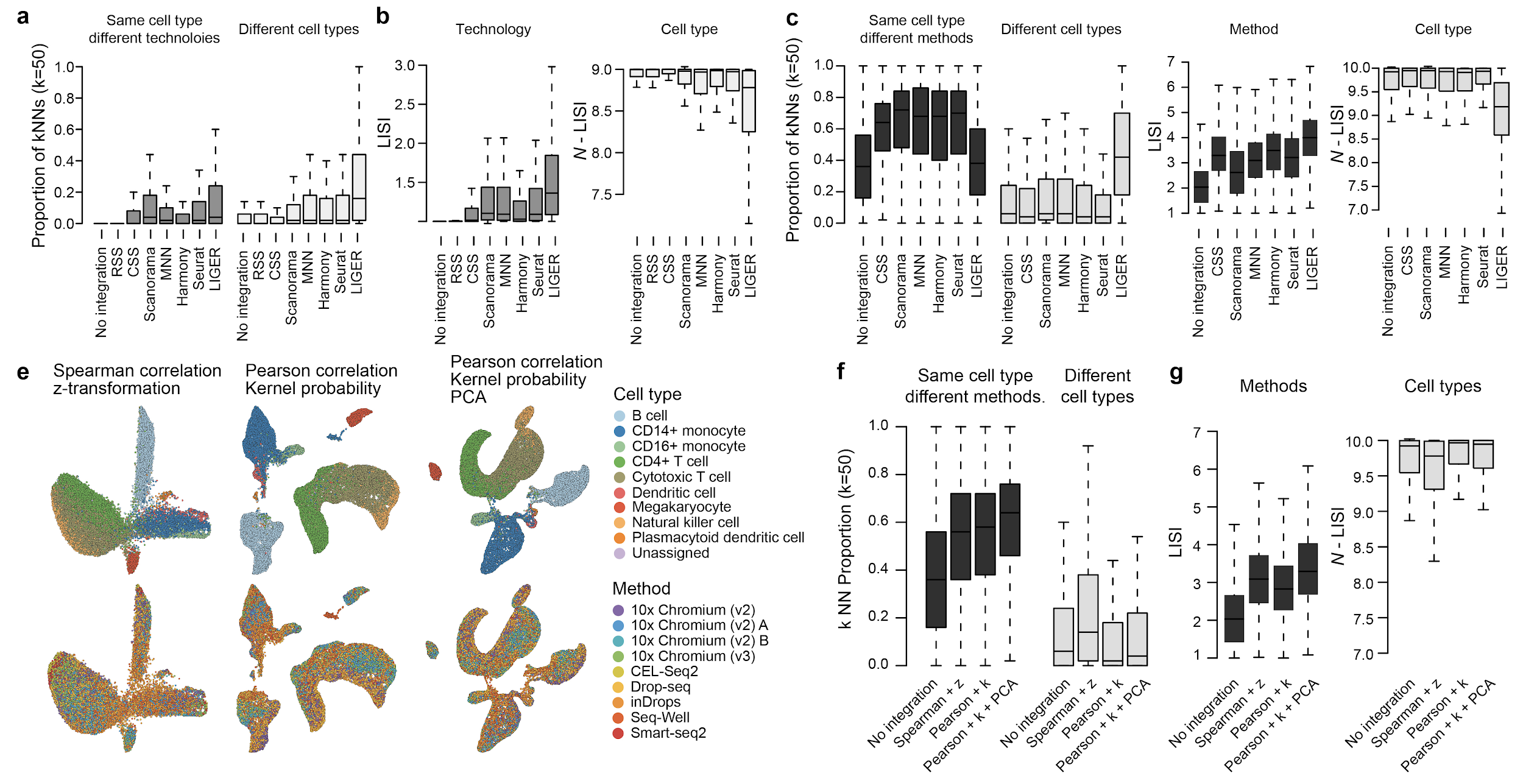


**Fig. S5. Benchmark of different integration methods on scRNA-seq technologies.** (a-b) Benchmark of different integration methods on the cerebral organoid scRNA-seq data set with three different technologies. (a) Proportion of neighbors of each cell which (left) share the same cell type but measured by different technologies, or (right) are of different cell types. Neighbors are defined as 50 cells with the shortest Euclidean distances with the cell in PCA (no integration) or different integration spaces. (b) LISI-based scores of technologies (left) and cell type annotation (right) with no or different integration methods being used. A higher score indicates better performance. (c-d) Benchmark of different integration methods on the PBMC scRNA-seq data set measured by different methods. (c) Proportion of neighbors of each cell which (left) share the same cell type but measured by different methods, or (right) are of different cell types. Neighbors are defined as 50 cells with the shortest Euclidean distances with the cell in PCA (no integration) or different integration spaces. (d) LISI-based scores of methods (left) and cell type annotation (right) with no or different integration methods being used. A higher score indicates better performance. (e) CSS-based UMAP embeddings of the PBMC scRNA-seq data set with different CSS parameters. The embeddings are colored by annotated cell types (upper row) and methods (lower row). (f-g) Similar to (c), but to benchmark different CSS settings for the PBMC scRNA-seq data set.
