# Supplementary material for: CSS: cluster similarity spectrum integration of single-cell genomics data
Source: Genome Biol. 2020 Sep 1;21:224. doi: 10.1186/s13059-020-02147-4 (PMC7460789; doi:10.1186/s13059-020-02147-4)
Supplement: Supplementary file 2 — Additional file 2. Review history. [file 13059_2020_2147_MOESM2_ESM.docx]

Review History

**First round of review**

**Reviewer 1**

**Were you able to assess all statistics in the manuscript, including the appropriateness of statistical tests used?**

Yes: I am a statistician so I can assess the statistical part of the manuscript.

**Were you able to directly test the methods?**

No

**Comments to author:**

In this work, He et al. developed a new computational method named CSS to integrate scRNA-seq data from multiple experiments. This is certainly a very important problem, and a number of methods have been developed very recently. CSS is an unsupervised reference-free method, meaning that it doesn't require external reference panel.

Overall, I feel the quality of this work is not up to the level of Genome Biology. Methodologically, there is not much novelty in CSS. It is very similar to RSS, only difference is that it is reference-free. The description of the method is unclear and not well justified. The real data results are not very convincing. They applied the method on several datasets and claimed to have better results than a few other methods. However, the comparison metrics used are not well designed.

Below are some specific comments.

I think the authors need to better emphasize the methodological contribution of this work, in particular the advancement over RSS.

The description of method is very vague. For example

-     It mentioned that RSS and CSS first select "highly variable genes". How variability is defined? By variance of raw counts, log-normalized-counts, or coefficient of variation?

-     CSS first cluster cell from different samples. How is that done? Does it use existing software like Seurat? Will this result have impact on the final results for CSS?

-     CSS computes the Spearman correlation between each cell and the cluster centroid in all samples, cover the correlation to z-scores, and then "standardize" the z-score. Why use Spearman correlation instead of Pearson correlation?

-     How exactly is the "standardization" done? What's the rationale behind such standardization?

-     It mentions that "standardization is done separately across clusters of different samples". I don't quite understand what this means. Does it mean CSS does standardization for the same cell type across samples? This brings up another question: after the initial clustering on different sample, how CSS align the cell types? Because this is unsupervised clustering, so that the cluster labels from different samples can be different, so it's important to align the cell types first.

-     The idea of normalizing batch effect based on correlation have been proposed on a recent paper "scBatch: batch-effect correction of RNA-seq data through sample distance matrix adjustment". Authors need to compare with that method to discuss the differences.

-     After computing the standardized correlation, it appears that each cell will have a similarity spectrum as a vector of length NxK, where N is the number of samples and K is number of cell types. After that, how can one use these values to group cells?

-     Overall, there are so many unknowns in the method description. It's very difficult for readers to follow and comprehend the ideas.

The real data results:

-     The definition of "neighboring cell" is not very clear: "Neighboring cells were defined as cells with the smallest Euclidean distances to the cell in the integrated space" - I don't know what exactly this means. So each cell will have one neighboring cell, since there can only be one cell with the "smallest" distance.

-     There are lots of hand-waving in the results, without providing concrete evidence. For example, claims like "Seurat v3 and LIGER suffered from an over-correction problem, where cells of different differentiation stages or of different brain regions were grouped together" need to be better justified. How can one know this? By just eyeballing the figures?

-     I think authors need to compare the results with SC3 and SC3+MNN. I think SC3 in general have better performance than Seurat.

-     I think using RSS-based cell type annotation as gold standard is in favor of CSS, since these two methods are similar. There are new datasets, for example, from this paper "A Bayesian mixture model for clustering droplet-based single-cell transcriptomic data from population studies". They have annotation from cell sorting, which should be a good reference dataset.

**Reviewer 2**

**Were you able to assess all statistics in the manuscript, including the appropriateness of statistical tests used?**

Yes: Statistics were clear

**Were you able to directly test the methods?**

Yes

**Comments to author:**

Here, He et al., present Cluster Similarity Spectrum (CSS), an unsupervised scRNA-seq data representation which the authors leverage to integrate single-cell genomic data. CSS represents a simple and elegant approach, taking inspiration from RSS, but using an intrinsic rather than extrinsic reference. Overall, this approach is well-presented and offers key advantages over other existing integration methods, for example, new data can be projected onto an existing reference atlas. In addition, CSS seems to avoid overcorrection issues that are frequently encountered by Seurat and LIGER - this is nicely demonstrated. However, the metrics for comparison to other methods are not clear and the benchmarking is currently not comprehensive, thus it's difficult to fully assess the capabilities of CSS. I have the following specific comments:

- A recent, thorough benchmarking of integration algorithms was presented here: https://www.biorxiv.org/content/10.1101/2020.05.22.111161v2.full To present the full potential and limitations of CSS, a similarly thorough benchmarking should be performed. For example, in this preprint, Scanorama appears to outperform other algorithms and clear, quantitative metrics and rankings are provides. Here, for CSS, the performance is not very clearly quantified, and Scanorama is not included for comparison. In fact, Seurat and Harmony are not the best performers according to this preprint. It would be much more transparent is the authors benchmark CSS against a much more comprehensive range of algorithms.

- The authors suggest that "CSS is able to resolve a developmental intermediate state". This claim should be validated using existing ground truth data.

**Authors Response**

**Point-by-point responses to the reviewers’ comments:**

Reviewer #1: In this work, He et al. developed a new computational method named CSS to integrate scRNA-seq data from multiple experiments. This is certainly a very important problem, and a number of methods have been developed very recently. CSS is an unsupervised reference-free method, meaning that it doesn't require external reference panel.

Overall, I feel the quality of this work is not up to the level of Genome Biology. Methodologically, there is not much novelty in CSS. It is very similar to RSS, only difference is that it is reference-free. The description of the method is unclear and not well justified. The real data results are not very convincing. They applied the method on several datasets and claimed to have better results than a few other methods. However, the comparison metrics used are not well designed.

*We thank the reviewer for the comment. We agree with the reviewer that CSS is somewhat similar to RSS, both to represent a cell by its transcriptomic similarity to certain reference transcriptomic profiles. However, as Reviewer 2 notes, “CSS represents a simple and elegant approach, taking inspiration from RSS, but using an intrinsic rather than extrinsic reference.” RSS calculates the similarities to the external reference profiles, which largely limits its application, as the proper and comprehensive external reference does not exist for most biological systems. CSS, on the other hand, avoids this problem by considering cell clusters in each sample/batch of the data set as references. As a cell likely shows higher similarity to cell clusters of the same sample, and similarities to clusters of different samples is likely affected by other technical variances (e.g. data quality), normalization is done for similarities to clusters of each sample separately, so that similarities to clusters of different samples do not interfere with each other. This is a critical difference from the RSS calculation. This concept, and perhaps more importantly, the computational tools associated with our manuscript, are both novel and useful. In addition, we introduced another similarity normalization method during the revision, which is to convert the similarities of a cell to clusters of one sample/batch into probabilities by applying the correlation kernel K_i=e^((-γ))*e^((γ*corr(X_i))). We did the comparison and have shown in Supplementary Figure 5, that using kernel probability transformation improved the CSS integration when applying to the PBMC scRNA-seq data set. More technical details are now added to the Methods.*

*We have been using CSS in multiple studies (e.g. https://doi.org/10.1101/2020.06.19.162032*

*and https://doi.org/10.1101/2020.07.24.219147) and we are very excited about its ease of use and high performance. Although it is conceptually simple, we believe that the method is very beneficial to the single cell genomic and developmental biology communities.*

Below are some specific comments.

*I think the authors need to better emphasize the methodological contribution of this work, in particular the advancement over RSS.*

The description of method is very vague.

*We thank the reviewer for the very valuable suggestions. We have largely expanded the Methods section for more detailed description of the methods and how exactly we used CSS in different data sets. Please see lines 356-418 and 471-640.*

For example

- It mentioned that RSS and CSS first select "highly variable genes". How variability is defined? By variance of raw counts, log-normalized-counts, or coefficient of variation?

*We thank the reviewer for the comment. In our analysis, highly variable genes were identified using the default vst method implemented in Seurat, which is based on local polynomial regression on log(variance) and log(mean) of feature values. Genes are ranked based on the resulting variance, and the top genes are selected as the highly variable genes. On the other hand, other methods can also be used to generate the gene list for CSS calculation, e.g. the union set of the most correlated genes to the top PCs, or the consensus variable gene list of different samples. In the CSS implementation in simspec, we provide the option to calculate CSS based on a customized gene list. We have added the description in the Methods section.*

- CSS first cluster cell from different samples. How is that done? Does it use existing software like Seurat? Will this result have impact on the final results for CSS?

*We thank the reviewer for the comment. In principle, any clustering method can be used to clustering cells from different samples. In this study and in the implementation in the simspec package, louvain clustering in Seurat is applied. We have now modified the Methods section to better clarify this point. The choice of the clustering methods definitely influences the results, as CSS represents each cell by its similarities to clusters obtained by this clustering. On the other hand, we compared the resulting k-nearest neighbor (kNN, k=50) networks obtained by CSS with different clustering resolution parameters for this clustering step, as well as the networks based on other integration methods, for the two-month-old cerebral organoid scRNA-seq data. It suggests that the kNN networks of CSS with different clustering are very similar to each other. We believe that this is an evidence that CSS is somewhat robust to the clustering. (Supplementary Figure 2)*

- CSS computes the Spearman correlation between each cell and the cluster centroid in all samples, cover the correlation to z-scores, and then "standardize" the z-score. Why use Spearman correlation instead of Pearson correlation?

*We thank the reviewer for the comment. In principle, any metric which can be used to quantify similarity between two transcriptomic profiles can be used, so both Spearman correlation and Pearson correlation are possible.In the presented analysis on cerebral organoid scRNA-seq data, we used Spearman correlation for its robustness to outliers. On the other hand, we also propose that in certain scenarios, e.g. when in a data set with discrete cell populations with very distinct gene expression signatures, Pearson correlation potentially provides higher sensitivity which can benefit the CSS integration. Therefore, we used Pearson correlation when analyzing the PBMC scRNA-seq data set. We have modified the Methods section accordingly to clarify these points more clearly.*

- How exactly is the "standardization" done? What's the rationale behind such standardization?

*We thank the reviewer for the comment. The rationale behind is that for one particular cell, its similarities to clusters of the same sample are potentially higher in general, because of the potential technical batch effect or individual variations between the sample and the others. Similar differences likely exist between other samples. These differences may then highlight the differences between different samples which makes proper integration less likely. To eliminate these differences, we separately scale similarities of one cell to clusters of different samples to obtain the normalized similarities of the cell to clusters in different samples separately. The normalized similarities represent the relative similarities of a cell to different clusters in the same sample, and we believe these relative similarities are more comparable across samples and more robust to batch effect or individual variations. We have modified the Methods section to better explain this rationale.*

*There are multiple ways to do “standardization” or normalization of similarities. In the analysis of cerebral organoid scRNA-seq data sets, we used z-transformation, to centralize and scale the values by their mean and standard deviation. We also proposed a different normalization method to convert the correlations into probabilities via correlation kernel: K_i=e^((-γ))*e^((γ*corr(X_i))). The resulting kernel vector of a cell is further normalized so that its sum becomes one. This method was used to analyze the PBMC scRNA-seq data set. We have modified the Methods section to describe these two different normalization methods. (Supplementary Figure 1)*

- It mentions that "standardization is done separately across clusters of different samples". I don't quite understand what this means. Does it mean CSS does standardization for the same cell type across samples? This brings up another question: after the initial clustering on different sample, how CSS align the cell types? Because this is unsupervised clustering, so that the cluster labels from different samples can be different, so it's important to align the cell types first.

*We thank the reviewer for the comment. As mentioned above, for each cell, the normalization of similarities is done separately for clusters of different samples. After normalization, the normalized similarities are concatenated. The resulting normalized similarity spectrum of each cell is a vector with length of n=∑_i n_iwhere n_iis the number of clusters in sample i. The numbers of clusters in different samples can be different and there is no alignment of cell type or cell clusters across samples.*

- The idea of normalizing batch effect based on correlation have been proposed on a recent paper "scBatch: batch-effect correction of RNA-seq data through sample distance matrix adjustment". Authors need to compare with that method to discuss the differences.

*We thank the reviewer for the comment. We agree with the reviewer that correlation-based representation for scRNA-seq data is not a new concept. Both scBatch and an earlier study (Li, et al. 2017 Nature Genetics) proposed to use a pairwise correlation matrix between cells to represent cells or assist the batch effect correction. This strategy, however, is limited by its scalability, and it is difficult to apply to the current scRNA-seq data with tens of thousands or even millions of cells measured. Instead of calculating pairwise correlation between cells, CSS only calculates correlations between cells and cell clusters which greatly increase the method scalability. The normalization step we introduce in CSS to correct for global differences on sample/batch level is principally simple, but easy to compute. Our benchmark of CSS and other integration methods also show the high and robust performance of CSS. We have modified the Discussion section to involve this comparison.*

- After computing the standardized correlation, it appears that each cell will have a similarity spectrum as a vector of length NxK, where N is the number of samples and K is number of cell types. After that, how can one use these values to group cells?

*We thank the reviewer for the comment. After computing the normalized similarities, each cell will be represented by a vector with length of n=∑_i n_iwhere n_iis the number of clusters in sample i. In other words, the data set will be represented by a N*n matrix where N is the total cell number. This data matrix can be used as the input of further analysis including different clustering algorithms, as well as other commonly used analysis in single cell genomics such as pseudotemporal ordering or pseudotime analysis. As n increases along with the sample number, additional dimension reduction (e.g. PCA) can be applied to the CSS matrix. The following clustering and other analysis can be then done using the resulting first PCs as the input. We used this strategy to analyze the PBMC scRNA-seq data set. We have modified the Methods section for better clarification. We have also provided an online vignette (https://github.com/quadbiolab/simspec/blob/master/vignette/vignette.md) to provide a better guideline of how to calculate CSS and how to use its output for other analysis. (Supplementary Figure 4)*

- Overall, there are so many unknowns in the method description. It's very difficult for readers to follow and comprehend the ideas.

*We thank the reviewer for the comment. We have modified the Methods section for better and more comprehensive description about technical details of CSS and our implementation in the simspec R package.*

The real data results:

- The definition of "neighboring cell" is not very clear: "Neighboring cells were defined as cells with the smallest Euclidean distances to the cell in the integrated space" - I don't know what exactly this means. So each cell will have one neighboring cell, since there can only be one cell with the "smallest" distance.

*We thank the reviewer for the comment. In brief, Euclidean distances were calculated between the cell and other cells in the data set in the data space after integration. Cells were ranked based on their distances to the cell of interest, and the first k cells (k=50 in our analysis) with the shortest distances were the neighboring cells of the cell of interest. This was done via the nn2 function in the RANN R package. Therefore, there are k (k=50 in our analysis) neighboring cells for each cell.*

- There are lots of hand-waving in the results, without providing concrete evidence. For example, claims like "Seurat v3 and LIGER suffered from an over-correction problem, where cells of different differentiation stages or of different brain regions were grouped together" need to be better justified. How can one know this? By just eyeballing the figures?

*We thank the reviewer for the comment. We have now introduced more quantitative metrics to assess performance of different integration methods. These metrics include the k-nearest neighbor (kNN) based metrics. For instance, the average proportion of kNNs of a cell which share the same cell type label but have different sample/batch labels can measure how well the mixing of cells of the same cell type but from different batches/samples, while the average proportion of kNNs which have different cell type labels measures the overall mistakes introduced by the integration. We also used Local Inverse Simpson's Index (LISI) based metrics, mostly the average LISI of batch/sample labels and cell type labels across all cells in the data set with different integration methods. In general, different metrics match with each other, suggesting that CSS provided as good performances as the other methods in batch effect correction, while showing the best performance in retaining cell type heterogeneity.*

*We realize that some of the metrics we used in the manuscript are not very straightforward, particularly those used to benchmark integrations of time course data sets. A part of the reason is that many of the existing metrics are designed for data sets with well-defined and discrete cell populations. However, a part of the aim of our manuscript was to show to the developmental biology community that CSS can be used to integrate scRNA-seq data from complex developing tissues. Therefore, we decided to focus most of the analysis on developing tissues (e.g. human retina) or the stem cell models for development (e.g. human cerebral organoids), where large numbers of cells at intermediate cell states are available as continuous trajectories and a very distinct and defined cell population is hardly seen.*

*Specifically for the over-correction problem of Seurat v3 and LIGER in the two-month-old cerebral organoid scRNA-seq data, we can not only see it from the embedding, but also in our quantitative benchmark, where Seurat v3 and LIGER performed much worse than other methods, not only CSS but also Scanorama, MNN and Harmony, in terms of mistakenly place cells of different cell types close with each other. On the other hand, they both indeed mixed cells from different organoids and batches together as suggested by both the UMAP embedding and the quantitative metrics. These two observations together suggest that these two methods suffered from an over-correction problem when being used in this data set. (Figure 2, Supplementary Figure 2)*

- I think authors need to compare the results with SC3 and SC3+MNN. I think SC3 in general have better performance than Seurat.

*We thank the reviewer for the comment. SC3 is a clustering algorithm for scRNA-seq data, not a data integration algorithm, which is a different task from the CSS representation we proposed. Principally it can replace the louvain clustering strategy that Seurat implements, which we currently used in the analysis and the simspec package. We chose Seurat for the clustering step for its scalability to large data and general widespread use in the scSeq community. As described above, we compared the resulting k-nearest neighbor (kNN, k=50) networks obtained by CSS with different clustering resolution parameters for this clustering step. The result suggests that CSS is robust to changes of the cell clustering on different samples. We think this is an evidence that even though SC3 can potentially provide better clustering results than the louvain clustering in Seurat, it won’t fundamentally affect the performance of CSS.*

*Furthermore, we have now included MNN in our analysis as another integration method for CSS to compare with, following the reviewer’s suggestion. In general, the performance of MNN is one of the best among all the integration methods in different data sets and scenarios we tested, better than the three methods we included at the beginning (Harmony, Seurat v3, LIGER). Meanwhile, our benchmark suggests that CSS performed comparably well or better than MNN, especially in avoiding mixing of different cell types. It gives us further confidence that CSS is a good strategy for scRNA-seq data integration.*

- I think using RSS-based cell type annotation as gold standard is in favor of CSS, since these two methods are similar. There are new datasets, for example, from this paper "A Bayesian mixture model for clustering droplet-based single-cell transcriptomic data from population studies". They have annotation from cell sorting, which should be a good reference dataset.

*We gratefully thank the reviewer for this important comment.*

*We agree with the reviewer that using the previous cell type annotation for the two-month-old cerebral organoid scRNA-seq data, which was mostly derived from RSS-based clustering, may introduce potential bias towards CSS. As there is no clear reference for a developmental system because cells are in various stages of transitions, we built an alternative cell type annotation for the data set which is integration-free. In brief, we applied clustering to the data set without integration, resulting in 25 clusters. Based on widely used regional identity markers including FOXG1, EMX1 and DLX2, prior regional identities of dorsal telencephalon, ventral telencephalon and non-telencephalon were assigned to cells in six, five and five clusters, respectively. It is worth mentioning that neurons with different regional identities are molecularly distinct from each other and form separated clusters. 1000 cells from each identity were randomly selected, and a LASSO regression model (with multinomial family) was fitted to predict regional identity of a cell given its expression values of the highly variable genes. The trained model was applied to each cell in the data set for the predicted regional identity. Next, we calculated a neural progenitor (NPC) score, defined as the average expression level of genes with higher expression level in NPCs than in neurons, and a neuron score, defined as the average expression level of genes with higher expression level in neurons, for each cell. A cell with higher NPC score than neuron score was considered as an NPC, and a cell with higher neuron score was considered as a neuron (Supplementary Fig. 2). For each cell, the combination of its predicted regional identity and estimated NPC/neuron identity was used as its alternative cell type annotation. Next, we benchmarked CSS and other integration methods using this alternative cell type annotation. The results are very consistent with the results based on the previous cell type annotation. The same strategy was also applied to the cerebral organoid data set with different experimental conditions, and the results are also consistent. (Supplementary Figure 2, Figure 4)*

*In addition, we applied CSS and other five integration methods (Scanorama, MNN, Harmony, Seurat v3, LIGER) also to the PBMC scRNA-seq data set from Broad Institute (Ding, et al. 2019, biorxiv, http://doi.org/10.1101/632216), which is commonly used in benchmark of integration methods. We showed that CSS performs comparably well as the other methods, especially in avoiding mixing of different cell types.*

Reviewer #2: Here, He et al., present Cluster Similarity Spectrum (CSS), an unsupervised scRNA-seq data representation which the authors leverage to integrate single-cell genomic data. CSS represents a simple and elegant approach, taking inspiration from RSS, but using an intrinsic rather than extrinsic reference. Overall, this approach is well-presented and offers key advantages over other existing integration methods, for example, new data can be projected onto an existing reference atlas. In addition, CSS seems to avoid overcorrection issues that are frequently encountered by Seurat and LIGER - this is nicely demonstrated. However, the metrics for comparison to other methods are not clear and the benchmarking is currently not comprehensive, thus it's difficult to fully assess the capabilities of CSS.

*We thank the reviewer for the encouraging overall assessment of our work as well as the critical suggestions. We have made additional analysis and substantial changes to the manuscript. More comprehensive benchmarking is done to compare CSS with existing integration methods and we believe that the manuscript is now greatly improved.*

I have the following specific comments:

- A recent, thorough benchmarking of integration algorithms was presented here: https://www.biorxiv.org/content/10.1101/2020.05.22.111161v2.full To present the full potential and limitations of CSS, a similarly thorough benchmarking should be performed. For example, in this preprint, Scanorama appears to outperform other algorithms and clear, quantitative metrics and rankings are provides. Here, for CSS, the performance is not very clearly quantified, and Scanorama is not included for comparison. In fact, Seurat and Harmony are not the best performers according to this preprint. It would be much more transparent is the authors benchmark CSS against a much more comprehensive range of algorithms.

*We thank the reviewer for this important comment. To more comprehensively and quantitatively assess performances of CSS and other integration methods in different data sets and scenarios, we introduced two groups of performance metrics. The first group is the k-nearest neighbor (kNN) based metrics. They are similar to what we presented initially but are now extended to all cells for all the datasets we used. We used the average proportion of kNNs of a cell which share the same cell type label but have different sample/batch labels to measure how well the mixing of cells of the same cell type but from different batches/samples. On the other hand, we used the average proportion of kNNs which have different cell type labels to measure the overall mistakes introduced by the integration. The second group of metrics is Local Inverse Simpson's Index (LISI) based metrics, mostly the average LISI of batch/sample labels and cell type labels across all cells in the data set with different integration methods. LISI was introduced in Korsunsky et al. 2019 and widely used in different benchmark work of integration methods including Tran, et al. 2020 Genome Biology and Luecken et al. 2020 biorxiv mentioned by the reviewer. The two metric groups show consistent results, suggesting that CSS provides comparably batch effect correction performances as other methods, while showing the best performance in retaining cell type heterogeneity.*

- The authors suggest that "CSS is able to resolve a developmental intermediate state". This claim should be validated using existing ground truth data.

*We thank the reviewer for this important comment. We agree with the reviewer that it is very important to provide ground truth evidence for this statement. We note that CSS can very nicely resolve well studied progenitor-to-neuron trajectories for the different brain regions (e.g. dorsal telencephalon) with intermediate states connecting radial glial progenitors, intermediate progenitors, early born/migrating neurons, and maturing neuron states. Additional analysis, focusing on characterizing and benchmarking the intermediate cells identified using different integration methods was done on the intermediate state between PSC and neuroectoderm (NEcto) which we reported in the previous submission, as well as the intermediate state between neuroepithelium (NEpith) and one-month-old cerebral organoids (Org1m). For intermediate cells identified based on each integration method, we estimated their molecular biases towards the start or end states as the average expression differences between markers of the start and end states. Our results suggest that CSS and MNN are the only two methods that identified intermediate cells in an unbiased manner, with CSS further outperformed MNN for both analysis on PSC-NEcto intermediates and NEpith-Org1m intermediates. Specifically for our characterization on the PSC-NEcto intermediate cells, we found further evidence that knocking out CYP26A1, the gene we highlighted as one marker gene we identified for the PSC-NEcto intermediate cells, can lead to reduced differentiation and growth arrest in response to retinoic acid in embryonic stem cells (Langton and Gudas, 2008 Developmental Biology). We have included these results and the additional citation to the Results section. (Supplementary Figure 3)*

*In addition, we did extra analysis by applying CSS and the other five integration methods including Scanorama, MNN, Harmony, Seurat v3 and LIGER to another developmental time course scRNA-seq data set of human retinal organoids and primary retina (Lu, et al. 2020 Developmental Cell), which includes 24 samples covering 20 different time points, including four retina organoids cultured for 24-59 days, 17 fetal retina samples aged between gastrulation week 9 to 27, one retina sample at postnatal day 8 and one adult retina. Using multiple kNN-based metrics, we showed that CSS successfully recovered continuity between cells from different time points without damaging the sample temporal orders, with minimal cost on mixing cells of different cell types. We further investigated two annotated intermediate cell states in the data set (AC/HC precursors, BC/Photo precursors), by estimating whether these intermediate cells keep their identity without over-mixing with their biological ancestry/progeny cell states or even other cell types. The result suggests that for both intermediate cell states, CSS integration preserved their within-group similarity better than other integration methods. We think these results further validate that CSS is able to resolve a developmental intermediate state. (Supplementary Figure 4)*

**Second round of review**

**Reviewer 1**

The authors has satisfactorily addressed all my concerns.

I want to thank the authors for their efforts in providing detailed and comprehensive responses. One recommendation for their future responses: I wish they can highlight the major changes in the revised manuscript in a different color, so that the reviewers can better locate the modifications. Of course they provided line numbers, but it's better to also highlight the text to make reviewers' job easier.

**Reviewer 2**

The authors have fully addressed my previous concerns. I particularly appreciate their application of CSS (in addition to 5 other integration methods) to the human retinal organoids and primary retina datasets.
